# Understanding Gastric GIST: From Pathophysiology to Personalized Treatment

**DOI:** 10.3390/jcm13143997

**Published:** 2024-07-09

**Authors:** Doru-Florian-Cornel Moga, Gabriela Vlădoiu, Anca-Maria Frățilă, Andreea-Alina Dan, Daniel Popa, Valentin Oprea

**Affiliations:** 1 Clinical Department of Surgery, Military Clinical Emergency Hospital Sibiu, 550024 Sibiu, Romania; cornel.moga@ulbsibiu.ro; 2 Department of Dental Medicine and Nursing, Faculty of Medicine, Lucian Blaga University Sibiu, 550024 Sibiu, Romania; 3 Clinical Department of Neurology, Emergency Clinical County Hospital of Sibiu, 550245 Sibiu, Romania; 4 Department of Dental Medicine, Military Clinical Emergency Hospital Sibiu, 550024 Sibiu, Romania; 5 Department of Radiology, Military Clinical Emergency Hospital Sibiu, 550024 Sibiu, Romania; at-radiologie@spitalmilitarsb.ro; 6 Department of Gastroenterology, Military Clinical Emergency Hospital Sibiu, 550024 Sibiu, Romania; danieliulian.popa@ulbsibiu.ro; 7 Clinical Department of Surgery, Military Clinical Emergency Hospital Cluj-Napoca, 400132 Cluj-Napoca, Romania; opreasv31@gmail.com; 8 Department of Surgery, Faculty of Medicine, Iuliu Hatieganu University Cluj-Napoca, 400012 Cluj-Napoca, Romania

**Keywords:** gastric, GIST, surgery, imaging, artificial intelligence

## Abstract

**Background:** Gastric gastrointestinal stromal tumors (GISTs) represent a subset of gastrointestinal tumors predominantly found in the stomach. Despite their rarity, these tumors carry significant implications for patient health and management. GISTs are potentially malignant tumors with unpredictable progression. They originate from the interstitial cells of Cajal, which are positioned between the intramural neurons and the smooth muscle cells of the digestive tract. These tumors are characterized primarily by mutations in the c-Kit gene, as well as other mutations such as those in the platelet-derived growth factor receptor alpha (PDGFRA) gene. **Methods:** Our comprehensive search across five databases initially yielded 2976 articles. After eliminating 197 duplicates, we screened the titles and abstracts of 2779 articles, excluding 2692 for not meeting the inclusion criteria. During the full-text screening, 16 more articles were excluded. Ultimately, 71 papers met the inclusion criteria and were included in our analysis. **Results:** Due to differences in study designs, inclusion criteria for patients, and reported outcomes, a meta-analysis was not conducted. The accurate diagnosis of GIST is established through histopathological examination and immunohistochemistry. Histopathologically, GISTs are classified into three main types: spindle cell, epithelioid, and mixed. The therapeutic management of GIST involves surgery, endoscopic treatment, and chemotherapy. **Conclusions:** The prognosis for GIST patients depends on various factors, including risk category, disease stage, applied treatments, and recurrence post-treatment. A significant recent advancement comes from artificial intelligence, which can be increasingly involved in both the diagnosis and treatment of this tumor.

## 1. Introduction

Gastric gastrointestinal stromal tumors (GISTs) represent a subset of gastrointestinal tumors predominantly found in the stomach. Despite their rarity, these tumors carry significant implications for patient health and management. Characterized by their unpredictable behavior and potential for malignant transformation, gastric GIST poses challenges in both diagnosis and treatment [1]. Gastric GISTs are relatively uncommon but represent a significant majority of all GIST cases [1,2].

Gastric GISTs can vary in size, with some presenting as small, incidental findings while others grow to substantial dimensions. GISTs are not classified as either benign or malignant but are rather stratified by their clinical risk of malignancy: very low, low, intermediate, or high [3].

Furthermore, research suggests that around 20% to 30% of gastric GISTs demonstrate malignant behavior, underscoring the importance of timely diagnosis and appropriate management strategies [2,4]. However, recent advances in molecular biology and targeted therapies have revolutionized our approach to managing this condition, offering renewed hope for patients and clinicians alike. In this article, we embark on a comprehensive exploration of gastric GIST, navigating through its epidemiology, pathophysiology, diagnostic strategies, and therapeutic interventions, while also spotlighting the latest breakthroughs shaping the landscape of GIST management.

## 2. Background

We conducted searches across multiple databases, including MEDLINE via PubMed, Web of Science, The Cochrane Library (specifically, the Cochrane Central Register of Controlled Trials-CENTRAL), Embase, and Scopus, utilizing a search strategy employing keywords related to our study topic. The keywords used were GIST, laparoscopy, laparoscopic, gastrointestinal stromal tumor, and gastric GIST. We included articles published between 2010 and 2024. Articles such as case reports, laboratory experiments, incomplete or ongoing studies, animal studies, letters, comments, editorials, and book chapters were excluded from our review.

## 3. Methods

**Figure 1:**
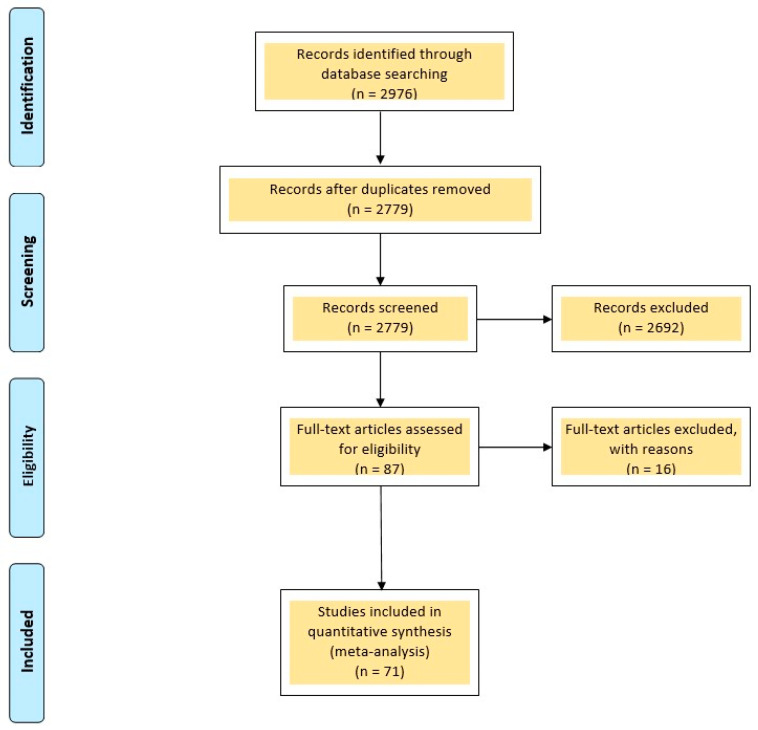
Selection process of the articles included in the review.

Our comprehensive search across these five databases yielded a total of 2976 articles. After removing 197 duplicate articles, we proceeded to screen the titles and abstracts of the remaining 2779 articles. During this screening process, 2692 articles were excluded for not meeting the inclusion criteria outlined in the methods section. Subsequently, 16 articles were further excluded during the full-text screening phase. Ultimately, 71 papers met the inclusion criteria and were included in our analysis. The selection process of these included articles is depicted in Figure 1.

## 4. Epidemiology and Risk Factors

Gastrointestinal stromal tumors pose unique challenges in clinical practice, given their rarity and diverse etiological underpinnings. While significant strides have been made in elucidating the molecular mechanisms driving GIST pathogenesis, continued research efforts are warranted to refine risk stratification strategies and optimize therapeutic interventions. A multidisciplinary approach, encompassing clinical genetics, oncology, and surgical expertise, is essential to ensure comprehensive care for individuals affected by GIST [4].

GIST constitutes a rare subset of gastrointestinal neoplasms, with an estimated incidence of 10 to 20 cases per million individuals annually. Geographic variability exists, with higher rates reported in certain regions. Data regarding the global distribution of GISTs underscore the importance of comprehensive epidemiological surveillance to discern regional trends and inform healthcare resource allocation [5]. However, accurately determining the incidence of GISTs is challenging due to the presence of mini-GISTs (asymptomatic tumors under 2 cm, often found incidentally during endoscopy) and micro-GISTs (typically under 1 cm, discovered incidentally during pathological examinations of resected specimens) [6].

While GISTs can manifest across all age groups, they predominantly affect adults, particularly those in their fifth to seventh decades of life. Notably, the median age at diagnosis is around 60 years. Moreover, there appears to be a slight male predominance in GIST incidence, though the underlying reasons necessitate further investigation [5,7].

The majority of GISTs originate in the stomach. However, these tumors can also arise in other segments of the gastrointestinal tract, including the small intestine, colon, and rectum. The anatomical distribution of GIST influences clinical presentation, diagnostic approaches, and therapeutic considerations [8].

In terms of risk factors most incriminated are:

Genetic Susceptibility: GIST pathogenesis is intricately linked to genetic alterations in proto-oncogenes encoding receptor tyrosine kinases, most notably KIT and platelet-derived growth factor receptor alpha (PDGFRA). While sporadic cases predominate, familial predisposition accounts for a minority of diagnoses, often associated with germline mutations in KIT or PDGFRA. Recognition of hereditary syndromes, such as familial GIST syndrome, necessitates comprehensive genetic counseling and surveillance strategies [9].

Environmental Exposures: Occupational exposures to certain chemicals, including vinyl chloride, have been implicated as potential risk factors for GIST development. Additionally, a history of abdominal radiation therapy, either for primary malignancies or benign conditions, confers an increased susceptibility to GISTs. Vigilance regarding environmental exposures and prior medical interventions is paramount in the clinical evaluation of individuals presenting with GISTs [10].

Underlying Medical Conditions: GISTs may occur concomitantly with specific hereditary syndromes, such as neurofibromatosis type 1 (NF1) and Carney–Stratakis syndrome. These syndromic associations necessitate comprehensive clinical assessment and genetic testing to elucidate underlying etiologies and inform management strategies. Furthermore, the presence of comorbid gastrointestinal disorders, such as gastrointestinal motility disorders or chronic inflammatory conditions, warrants consideration in the evaluation of GIST risk factors and therapeutic approaches [11].

## 5. Pathophysiology

Gastric GISTs originate from the interstitial cells of Cajal (ICCs) or their precursors, specialized pacemaker cells distributed throughout the gastrointestinal tract responsible for regulating peristalsis and gut motility [6]. The majority of GISTs have a gain-of-function mutation in either KIT (70%) or PDGFRA (10–15%), and some (nearly 15%) may have other mutations in BRAF, RAS family genes, and NF1, and alterations in the SDH (succinate dehydrogenase; complex III in the mitochondrial electron transport system) complex or in NRTK translocation. These mutations and alterations are mutually exclusive in primary GISTs [6].

The hallmark of gastric GIST pathophysiology lies in the dysregulation of receptor tyrosine kinase (RTK) signaling pathways, primarily involving the KIT (CD117) and platelet-derived growth factor receptor alpha (PDGFRA) genes. These mutations lead to constitutive activation of downstream signaling cascades, including the RAS-RAF-MEK-ERK and PI3K-AKT-mTOR pathways, resulting in uncontrolled cellular proliferation and inhibition of apoptosis. In gastric GISTs, aberrant activation of KIT and PDGFRA promotes tumor growth and progression by stimulating cell cycle progression, enhancing angiogenesis, and facilitating evasion of immune surveillance mechanisms. Additionally, dysregulated signaling within the tumor microenvironment promotes tumor invasion and metastasis, contributing to the aggressive behavior observed in some cases [6,12].

Histologically, gastric GISTs exhibit a spectrum of morphological patterns, including spindle cell, epithelioid, or mixed phenotypes. Immunohistochemical analysis typically reveals strong positivity for KIT (CD117) and CD34, aiding in the diagnosis of gastric GIST and distinguishing them from other mesenchymal neoplasms. The clinical course of gastric GIST varies widely, ranging from indolent, localized lesions to aggressive, metastatic disease. Prognostic factors such as tumor size, mitotic rate, and histological grade play a crucial role in determining disease outcomes and guiding therapeutic decision-making. The advent of tyrosine kinase inhibitors (TKIs), notably imatinib mesylate, has revolutionized the management of advanced gastric GIST by targeting the underlying oncogenic signaling pathways driving tumor growth. However, acquired resistance to TKIs remains a significant challenge in the management of metastatic disease, necessitating the development of novel therapeutic strategies and personalized treatment approaches [9,13,14].

In summary, gastric GISTs represent a paradigm of oncogenesis driven by dysregulated RTK signaling, with implications for diagnosis, prognosis, and therapeutic intervention. Understanding the intricate pathophysiological mechanisms underlying gastric GISTs is essential for optimizing patient care and advancing treatment strategies in the era of precision oncology.

## 6. Clinical Presentation and Diagnosis

Regarding the clinical manifestation of GISTs, there is a large panel of symptoms, with a mean interval of 6 months between the first clinical symptom and the diagnosis [5]. GISTs range in size from a few mm to 35 cm, with a median size between 5 and 8 cm [15].

Small tumors are often discovered incidentally during surgery, endoscopy, or imaging studies performed for other reasons. Common symptoms include anemia, weight loss, gastrointestinal bleeding, abdominal pain, and effects related to a mass. Patients may also present with acute abdomen, obstruction, perforation, rupture, or peritonitis. Other symptoms can include nausea, vomiting, and abdominal distension [1,16]. Symptomatic tumors often cause a mass effect, leading to early satiety or obstruction [17].

About 50% of GISTs have metastasis at presentation. GISTs metastasize usually to the liver (65%) and peritoneum (21%) [1,18]. Other less common locations include lung, bone, brain, pleura, and lymph nodes [19].

The most frequent cancers associated with GISTs are gastrointestinal carcinomas, followed by extra-intestinal tumors. These include lymphoma/leukemia, as well as carcinomas of the prostate, breast, kidney, lung, and female genital tract. Other associated cancers are carcinoid tumors, soft tissue and bone sarcomas, malignant melanomas, and seminomas [20].

Imaging modalities play a crucial role in the comprehensive evaluation of gastric GIST, facilitating diagnosis, staging, and treatment planning. A multimodal approach, integrating CT, EUS, MRI, and PET imaging, allows for precise localization, characterization, and assessment of tumor extent, guiding therapeutic decisions and optimizing patient outcomes [21].

GISTs have a large range of radiological appearances based on the imaging technique, the site of origin, type of growth, and size.

Conventional abdominal radiographs and barium series currently have a limited role in diagnosing these tumors, by showing a non-specific lesion exerting a mass effect on the gastric air bubble and displaying features of intraluminal space-occupying lesion that appear as a filling defect with well-defined margins, that form an obtuse or right angle with the gastric wall, with a homogenous or inhomogeneous structure displaying focal ulcerations, internal erosions, calcifications, or fistulous tracts [22].

Abdominal ultrasound (US) can depict a large tumor with a heterogeneous structure, frequently having areas of necrosis, and floating inner echoes from debris, but the organ of origin is often difficult to assess. However, US, and especially contrast-enhanced US-CEUS can display hepatic metastases quite well, as hypoechoic lesions relative to the liver parenchyma, which enhance mostly peripherally. Endoscopic ultrasound is usually used to detect small tumors (<2 cm) because it is able to clearly delineate the gut wall layers and it can also be used to biopsy the lesion [22].

These tumors usually arise from the outer muscular layer of the gastric wall, and thus, computed tomography (CT) can fairly well assess the involvement of the lumen, the wall, and also the serosa. They are often seen as well-defined exophytic masses with different densities based on their inner structure in keeping with the size (areas of necrosis in large tumors and sometimes calcifications which are rare and can be mottled or quite extensive), extending outwards from the gastric wall. Small tumors, less than 5 cm, are often benign and usually have a homogeneous structure both on unenhanced and enhanced scans, although they can display central areas of hypoenhancement due to areas of hypocellularity, cystic degeneration, necrosis, hemorrhage, and ulcerations, although these features are more often seen in larger tumors (>6 cm) which have a higher propensity of being malignant [22,23,24].

MRI offers morphological findings comparable to those of CT and is particularly useful for evaluating large tumors. It effectively characterizes the extent of the lesion and its internal structure, with the tissue component appearing hypointense on T1 and hyperintense on T2, showing strong contrast uptake. Hemorrhages display varying intensities depending on the stage of the bleed. Additionally, MRI allows for quantitative measurements, such as apparent diffusion coefficient (ADC), perfusion parameters, and degree of enhancement, providing better information about treatment response. MRI is also more sensitive than CT in detecting hepatic metastases [22,24].

PET-CT allows visualization of viable tumor tissues with glucose metabolism, which helps in differential diagnosis from non-GIST tumors. It is also very useful in characterizing metastases and pathological lymph nodes, but false positive lesions can occur due to increased FDG uptake by inflammatory lesions, especially on post-surgical follow-up exams [22,24].

The diagnosis of GISTs is based on a combination of imaging findings and pathology [9].

Gastric GISTs can present with endophytic, exophytic, or mixed patterns, with the exophytic pattern being the most common [18,25]. Endo-sonographically, GISTs are classified into four subtypes based on their relation to the muscularis propria: Type I tumors protrude into the digestive lumen with a narrow attachment, Type II tumors protrude with a broad connection, and Type III tumors are centrally located on the gastric wall. Type IV: tumors extend outward, protruding into the serosa of the gastric wall [26].

There is no general consensus on the necessity of a preoperative histological diagnosis for resectable, intramural, clinically significant tumors of the gastrointestinal tract [27]. The diagnostic rate of GIST through EUS-FNA varies between 62.0% and 93.4%. Depending on the tumor diameter, the diagnostic rate stands at 71% for tumors sized between 1 to 2 cm, 86% for those measuring 2 to 4 cm, and reaches 100% for tumors larger than 4 cm [28]. Traditional endoscopic biopsy poses challenges for non-ulcerated tumors. In cases with ulcerated lesions, jumbo forceps sampling can provide a diagnostic solution [17].

A preoperative biopsy is typically not advised in cases where there is a strong suspicion of GIST and the lesion is deemed resectable [29]. Immediate laparoscopic or open excision may be considered on a case-by-case basis, particularly if surgery entails minimal morbidity [30]. When a lesion is deemed suspicious, resectable, or operable, preoperative endoscopic biopsy is unnecessary [18,31]. For lesions larger than 5 cm or those associated with symptoms such as bleeding or pain, surgery is recommended even in the absence of a confirmed diagnosis [32].

When the tumor measures less than 2 cm, deciding on surgery becomes less definite. The clinician should consider performing periodic endoscopic and radiographic surveillance. If there is a trend towards increased or high-risk features on EUS (unclear borders, cystic degeneration, ulceration, hemorrhage, and heterogeneity), curative surgery must be considered whenever possible [32]. If a submucosal tumor is not identified as a GIST or another malignant condition surgical resection is not required, as most non-GIST submucosal tumors, such as leiomyoma, ectopic pancreas, or schwannoma, are benign [33].

The potential diagnosis of gastric GIST is typically confirmed immunohistochemically using anti-CD117 (c-kit) antibodies, as nearly all GISTs (approximately 95%) are positive for c-kit [34].

## 7. Treatment Options

### 7.1. Management of Local/Locoregional Disease

Surgery is the primary and only treatment that offers a permanent cure for localized primary GIST [6,35].

**Figure 2:**
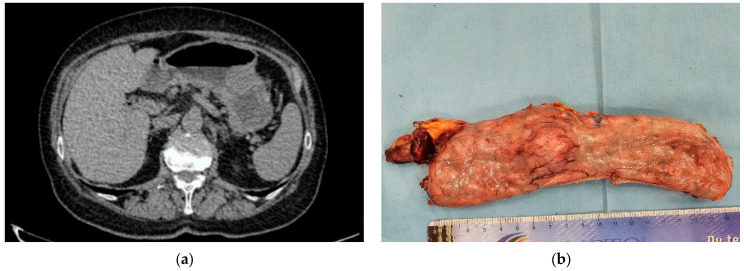
(**a**) CT aspect of gastric GIST. (**b**) Wedge resection for an endophytic gastric GIST.

For patients with localized or potentially resectable GIST lesions, the aim is to achieve a complete surgical resection, with an intact capsule [27,35] (Figure 2a,b). Segmental or wedge resection of the stomach is accepted, thus, aggressive and more extensive surgery to remove unaffected tissue is unnecessary [31] because a wide surgical margin does not improve outcome [4] or decrease recurrence [3]. Additionally, lymphadenectomy is usually not required given the low incidence of nodal metastases [30,36], unless the lymph nodes are enlarged [32].

Since GISTs are very friable, it is crucial to avoid injuring the tumor capsule during surgery, as tumor rupture is a significant adverse prognostic factor. Tumor rupture is characterized by spillage or fracture within the abdominal cavity, piecemeal resection, laparoscopic or open incisional biopsy, gastrointestinal perforation into the abdominal cavity, blood-tinged ascites, or microscopic transperitoneal infiltration into adjacent structures [17,30,35].

GIST may develop in any part of the stomach. The anterior wall of the stomach is more easily accessible than the posterior wall, and the greater curvature is easier to reach than the lesser curvature [8,34]. Non-anatomic resection (wedge or disk) of the gastric lesion should be completed in such a way as to prevent gastric wall deformity and preserve gastric function [3,37]. A partial gastrectomy or even total gastrectomy may be required for large tumors or those encroaching on the esophagus or pylorus [15,38].

As experience has accumulated, some of the criteria and recommendations have changed. The 2007 NCCN guidelines initially recommended laparoscopic intervention solely for tumors with a diameter of less than 2 cm [25]. However, this indication was later extended to encompass tumors up to 5 cm, particularly for lesions restricted to the anterior gastric wall. In recent times, GIST tumors smaller than 2 cm in diameter are usually monitored closely, while tumors larger than 10 cm may be considered suitable for a minimally invasive approach (MIS) in specialized centers [25,39].

Various surgical approaches are recommended based on the size and locations of the tumors [31]. Mainstream GIST resection techniques include open surgery and minimally invasive procedures such as laparoscopy, robotic surgery, and endoscopy [38].

**Figure 3:**
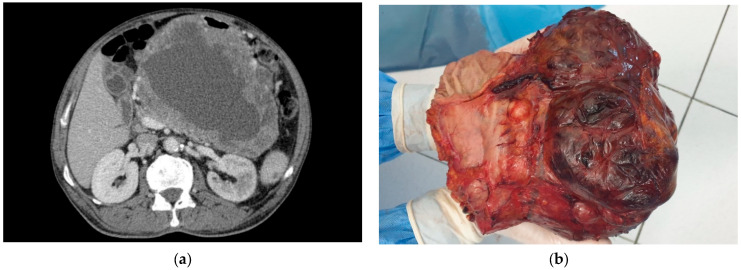
(**a**) Large gastric GIST. (**B**) Subtotal gastrectomy for large gastric GIST.

Open surgery has traditionally been the standard of care and remains the preferred option for large resectable tumors [25] (Figure 3a,b). En bloc resection, which includes surrounding tissues, is essential for GISTs that have invaded or adhered to adjacent organs or structures [32].

MIS has progressively supplanted open surgery owing to its benefits, including reduced intraoperative blood loss, decreased risk of complications, faster postoperative recovery [40,41], and improved cosmetic outcomes compared to open surgery, while maintaining similar oncologic prognoses [6]. Advancements in operative tools and techniques have expanded the options available for tumor resection, providing more choices than ever before [42]. Appropriate pre-operative selection of patients for MIS can improve outcomes and avoid undesirable results [43].

Laparoscopic surgery offers multiple options, including total, subtotal, distal, gastric stump, or proximal gastrectomy, as well as wedge resection, transgastric resection, seromuscular dissection, and laparoscopic intragastric submucosal dissection [44]. Among these options, laparoscopic wedge resection is commonly performed for gastric GISTs and has emerged as the primary treatment for small- to medium-sized gastric submucosal tumors. In cases of smaller tumors, intraoperative endoscopy is useful for guiding the surgeon to the lesion’s location, ensuring adequate oncological margins, and maintaining the patency of the remaining gastrointestinal lumen [6,45].

Robotic surgery facilitates a safer and more accessible minimally invasive treatment option for gastric GISTs larger than 5 cm, particularly those located in challenging surgical areas such as the juxtacardial, lesser curvature, and antropyloric regions [25]. The robotic approach may be advantageous compared to laparoscopic surgery due to the technical enhancements it brings [34].

However, MIS may not be suitable for tumors necessitating complex procedures like multivisceral resection [44]. Additionally, a laparoscopic or robotic approach is not recommended for patients with large tumors due to the risk of tumor rupture [30]. Conversely, the potential benefits of MIS for very large lesions should be carefully weighed, as an abdominal incision may ultimately be required to remove the specimen [25].

Endoscopic techniques offer alternative treatment options, particularly for small tumors. Type I GISTs can undergo endoscopic enucleation, with Type II lesions also being potential candidates. Types III and IV may benefit from various endoscopic treatment modalities, including submucosal dissection, excavation, full-gross resection, tunneling resection, and laparoscopic and endoscopic cooperative surgery [26]. Even for gastric GISTs with a diameter of 5 cm, endoscopic resection may be technically feasible in select cases. It not only yields short-term outcomes comparable to laparoscopic resection but also provides the benefits of swift postoperative recovery and cost-effectiveness [16].

### 7.2. Management of Advanced/Metastatic Disease

The percentage of patients deemed ineligible for resection and diagnosed with metastatic or unresectable disease has been reported to range from 13% to 50% [46].

It is necessary, in some cases of locally advanced GIST, to remove neighboring organs and perform a surgical en bloc excision [31]. Whenever feasible, it is best to avoid extended anatomic resections and complex multivisceral resections, aiming for function-preserving surgery. For tumors deemed unresectable due to significant morbidity, neoadjuvant therapy is the preferred approach. Confirming a GIST diagnosis and genetic examinations necessitate a biopsy. In cases of unresectable tumors or metastatic disease, initiating tyrosine kinase inhibitor (TKI) treatment is advisable [30,32].

Pre-operative treatment with imatinib should be considered under CT monitoring performed, with the possibility of surgical management when maximum response is achieved [14,18]. The preoperative treatment period typically lasts between 6 to 12 months and should not surpass 1 year. Early evaluation of imatinib’s efficacy, usually within a month of initiation, is crucial. Imatinib therapy should continue if no disease progression is evident based on enhanced CT scan findings. Active imatinib treatment not only reduces tumor size but also diminishes vascularity, potentially enhancing surgical safety and preventing intraoperative rupture [6]. Imatinib administration can be halted just before surgery and resumed once the patient can tolerate oral medications [47].

Surgery for metastatic or recurrent GISTs is a matter of debate, with careful case selection being paramount. It may benefit patients whose tumors are responding to adjuvant therapy, those showing limited focal progression, or those seeking palliative treatment [48].

**Figure 4:**
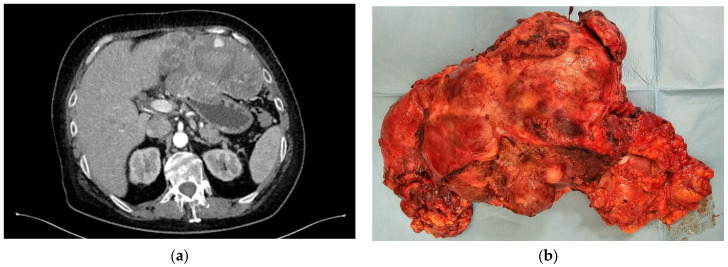
(**a**) Invasive gastric GIST in left hepatic lobe and diaphragm; tumor progression under Imatinib treatment; patient with severe obstructive symptoms. (**b**) Total gastrectomy, atypical left hepatic lobe resection, and debulking.

In certain instances, surgical intervention may be feasible for unresectable, advanced, and recurrent GISTs. If there is a favorable response to imatinib and the disease is effectively managed, surgery may be considered. This encompasses scenarios such as initially unresectable GISTs that have responded well to imatinib and are now resectable, locally advanced GISTs due to secondary resistance, low-volume stage IV disease, or cases necessitating palliative surgery to manage symptoms like bleeding or obstruction [32] (Figure 4a,b).

## 8. Advancements in Targeted Therapy—Prognosis and Follow-Up

**Adjuvant therapy**

Important prognostic factors for GISTs include the mitotic rate, tumor size, and tumor site, with gastric GISTs generally carrying a more favorable prognosis compared to those in the small bowel or rectum. Tumor rupture is an additional adverse prognostic factor and should be documented irrespective of whether it occurred before or during surgery. High-risk factors for recurrence in patients with stromal tumors include tumor size larger than 5 cm, mitotic count exceeding 5 counts per 50 high-power fields (5/50 HPF), tumor rupture, postoperative recurrence risk exceeding 50%, and the location of the tumor [14,49] (Table 1).

Patient age, particularly those under 50 years, and female gender have been significantly linked to a more favorable prognosis in GIST [50].

The recommendations provided by both the National Comprehensive Cancer Network (NCCN) and the European Society for Medical Oncology (ESMO) advocate for adjuvant imatinib therapy lasting at least 3 years in patients deemed to have significant or high risk of recurrence, while it is not advised for those at low risk [31,51].

Additionally, patients receiving neoadjuvant imatinib require postoperative adjuvant therapy for a duration of 3 years, aligning with the recommended period for high-risk GIST cases [32].

As of 2021, there have been 75 drugs undergoing clinical trials for GIST, with 5 drugs—imatinib mesilate, sunitinib, regorafenib, ripretinib, and avapritinib—approved for marketing. Among these, imatinib and sunitinib are widely available. All five drugs belong to the category of tyrosine kinase inhibitors (TKIs). Imatinib, avapritinib, larotrectinib, and entrectinib are utilized in first-line therapy, while sunitinib and dasatinib are employed in second-line therapy. Regorafenib and ripretinib, on the other hand, are, respectively, employed in third- and fourth-line therapy [52].

Treatment based on genetic information has been established, and in the future, emerging strategies featuring newly developed TKIs, molecularly targeted drugs, and immunotherapy may hold significant promise in GIST treatment [32]. The selection of optimal targeted therapy should be guided by genomic profiling [17].

**Follow-up and recurrence**

The objective of follow-up in GISTs is to identify subclinical disease while the tumor burden is still minimal [27].

Gastrointestinal stromal tumors (GISTs) can recur after treatment, even following surgical resection and adjuvant therapy. Recurrence in GISTs typically manifests due to incomplete surgical resection, microscopic residual disease, or acquired resistance to tyrosine kinase inhibitors (TKIs) like imatinib [27]. The risk of recurrence depends on several factors, including tumor size, mitotic rate, and location. High-risk GISTs are more prone to recurrence compared to low-risk ones. Management of recurrent GISTs often involves a combination of surgical resection, TKI therapy, and in some cases, enrollment in clinical trials exploring newer treatment modalities. Regular surveillance with imaging studies and clinical assessments is crucial for detecting recurrence early and initiating appropriate therapeutic interventions [28,29].

High-risk patients typically undergo regular follow-up appointments, which include abdominal CT scans or MRIs every 3–6 months for the initial 3 years during adjuvant therapy. These appointments involve closer clinical monitoring to manage potential side effects. Following the discontinuation of adjuvant therapy, imaging is performed every 3 months for 2 years, then every 6 months until 5 years post-adjuvant therapy cessation, with annual monitoring recommended thereafter for an additional 5 years [30].

The requirement for routine follow-up in low-risk tumor cases remains unclear. However, if deemed necessary, this may involve abdominal CT scans or MRIs every 6–12 months over a span of 5 years [30]. Although very low-risk GISTs probably do not mandate regular follow-up, there is still some risk involved. Given the consideration of X-ray exposure, particularly in low-risk GIST cases, abdominal MRI presents itself as a suitable alternative [30].

## 9. Results

Due to differences in study designs, inclusion criteria for patients, and reported outcomes, a meta-analysis was not conducted. Instead, the final conclusions of the reviewed articles are summarized in Table 2.

Artificial intelligence (AI) can play several valuable roles in the management of gastric gastrointestinal stromal tumors (GISTs), contributing to improved diagnosis, treatment planning, and patient outcomes. There are some ways AI can help clinicians regarding gastric GIST:

Diagnostic Augmentation: AI algorithms, trained on vast datasets of medical imaging studies, can serve as valuable aids to radiologists and gastroenterologists in interpreting imaging findings related to gastric GISTs. By analyzing features such as tumor morphology, enhancement patterns, and growth kinetics on CT, MRI, and endoscopic images, AI can provide quantitative assessments and differential diagnoses, assisting in accurate tumor detection and characterization [68].

Risk Stratification Refinement: AI-based predictive models, incorporating clinical, pathological, and molecular data, offer refined risk stratification for patients with gastric GISTs. By leveraging machine learning algorithms, these models can integrate complex datasets to identify subtle prognostic indicators and predict individualized risk profiles, aiding in treatment decision-making and patient counseling [69,70].

Treatment Optimization: AI algorithms, trained on comprehensive datasets encompassing treatment outcomes and molecular profiles of gastric GISTs, can guide treatment selection and optimization. By analyzing factors such as tumor mutational status, expression of therapeutic targets (e.g., KIT and PDGFRA), and treatment response patterns, AI can predict the efficacy of targeted therapies such as tyrosine kinase inhibitors (TKIs), facilitating personalized treatment strategies and minimizing unnecessary interventions [69,70].

Surgical Precision: AI-powered image analysis tools enable precise surgical planning and execution for gastric GISTs. By generating detailed 3D reconstructions of tumor anatomy from preoperative imaging, AI assists surgeons in delineating tumor margins, identifying critical structures, and selecting optimal surgical approaches. Additionally, real-time intraoperative guidance provided by AI-based navigation systems enhances surgical precision and minimizes the risk of complications [70].

Prognostic Advancement: AI-driven prognostic models, integrating diverse clinical and molecular parameters, offer refined prognostic assessments for gastric GIST patients. By analyzing large-scale datasets and identifying subtle prognostic factors, AI enhances risk prediction accuracy, enabling clinicians to stratify patients more effectively and tailor surveillance strategies accordingly [70,71].

In summary, AI holds immense potential to revolutionize the management of gastric GISTs by augmenting diagnostic accuracy, refining risk stratification, optimizing treatment selection, enhancing surgical precision, and advancing prognostic assessments. Continued research and clinical validation are essential to integrate AI-driven technologies seamlessly into routine clinical practice, ultimately improving patient outcomes and quality of care in the management of gastric GISTs.

## 10. Limitations of the Study

We consider that the limitations of this article are that the keywords were insufficient or partially conclusive. At the same time, we consider it necessary to mention the fact that articles were excluded that may have added value to this review. The aim of this review was to summarize the current state of research and at the same time to add new information regarding gastric GIST treatment. For this reason, we consider it necessary to continue research in the presented field.

## 11. Conclusions

In conclusion, gastric GISTs present complex challenges, but advancements in diagnosis, surgery, and targeted therapies offer hope. The article provides up-to-date information about GIST, aiding practitioners who encounter this pathology by reviewing its etiology, pathophysiology, clinical symptomatology, and current therapeutic approaches. Ongoing research is needed to address remaining challenges and improve outcomes. Collaboration among clinicians, researchers, and patients is essential in this evolving field.

## Figures and Tables

**Table 1:** Primary gastric GIST risk assessment guidelines (adapted after [31]).

Tumor Parameters		Risk of Progression (%)
Mitotic Index	Size (cm)	
≤5	≤2	No
	>2 to ≤5	Very low (1.9)
	>5 to ≤10	Low (3.6)
	>10	Moderate (10)
>5	≤2	No
	>2 to ≤5	Moderate (16)
	>5 to ≤10	High (55)
	>10	High (86)

**Table 2:** Conclusions of the reviewed articles. The Power of AI in the Management of Gastric GISTs.

Author, Name, Year of the Study	Cohort Size (n)	Summary of Findings
Shannon et al., 2021 [7]	949	Surgery requires careful patient selection in octogenarians to minimize postoperative mortality.
Sorour et al., 2014 [1]	92	The incidence of GISTs is increasing, and patient prognosis is closely tied to tumor size and the completeness of resection.
Hu et al., 2016 [53]	176	Laparoscopic surgery is both safe and effective, especially for GISTs sized between 2 and 8 cm, offering advantages over open surgery.
Soreide et al., 2016 [54]	13,550	Gastric GISTs exhibit a relatively equal distribution between genders, with the typical onset occurring around the age of 60.
Supsamutchai et al., 2018 [55]	68	A high mitotic index count is a crucial determinant of recurrence or metastasis in gastric GISTs.
Loureiro et al., 2016 [2]	15	Laparoscopic resection of GISTs remains a safe and acceptable technique, even for tumors larger than 5 cm.
Costache et al., 2018 [5]	42	Larger tumors may require the use of traditional surgical techniques, with limited intraoperative manipulation being key to reducing recurrence risk.
Stanciulea et al., 2020 [34]	25	MAS approach of gastric GISTs is associated with lower morbidity, compared to open surgery.
Hanayama et al., 2022 [37]	40	LECS is a safe and effective surgical procedure that yields good clinical outcomes for gastric intraluminal and intramural GISTs that are 50 mm or smaller in diameter.
Sharma et al., 2021 [56]	2418	Gastric GISTs are not a homogeneous disease; different regions of the stomach have varying risks of developing specific GIST subtypes, each with unique drug sensitivity and resistance profiles.
Zhao et al., 2022 [40]	53	Hand-assisted laparoscopic surgery (HLS) for gastric stromal tumor resection provides several benefits, including reduced operation times, minimal invasiveness, and optimal preservation of gastric function, especially for patients with GISTs located in the gastric cardia.
Seo et al., 2023 [8]	30	The surgical outcomes of SILS (single incision laparoscopic surgery) may surpass those of CLS (conventional laparoscopic surgery).
Park et al., 2022 [13]	915	Laparoscopic resection for patients with gastric GIST (even tumors greater than 5 cm or in unfavorable locations) can result in a lower complication rate and shorter hospital stays compared to open surgery, without compromising oncological safety.
Liu et al., 2017 [49]	301	Patients with gastrointestinal bleeding caused by GIST have shorter recurrence-free survival and overall survival.
Kim et al., 2014 [4]	406	The recurrence rate of gastric GISTs after curative resection was lower than that reported in other Western studies.
Nishida et al., 2021 [6]	52	The treatment approach for gastric GISTs smaller than 2 cm may need reconsideration due to their indolent nature and the presence of competing risks in these patients.
Ceccarelli et al., 2021 [25]	105	Robotic surgery, where accessible, offers a safer and more convenient minimally invasive option for treating gastric GISTs larger than 5 cm, particularly those situated in challenging surgical sites like the juxtacardial region, lesser curvature, and antro-pyloric regions.
Khan et al., 2022 [17]	10,833	Tumors larger than 5 cm in size and of high histologic grade demonstrated the most significant impact on survival.
Ongprakobkul et al., 2020 [57]	22	The majority of patients were over 40 years old, with gastrointestinal bleeding and abdominal pain being the predominant presenting symptoms. Surgery was the preferred treatment option.
Spiridon et al., 2021 [58]	46	The ghrelin axis may impact GISTs carcinogenesis by activating GHS-R.
Ceausu et al., 2021 [29]	57	Gastric GISTs exhibit the largest dimensions. Upper digestive endoscopy achieved close to 100% sensitivity for selected indications in upper digestive sites. Regardless of location, the CT scan was identified as the most sensitive investigative tool overall.
Rodriquenz et al., 2016 [20]	128	The elevated occurrence of second tumors implies the potential involvement of an unidentified shared molecular mechanism; however, given the brief interval between GIST diagnosis and the emergence of secondary neoplasms, meticulous follow-up, especially during the initial 3 years post-diagnosis, is warranted.
Severino et al., 2016 [59]	50	Laparoscopic resection for large GISTs is safe, technically feasible, and does not adversely affect the oncologic course or compromise oncological safety.
Chen et al., 2016 [60]	563	Laparoscopic resection for gastric GISTs demonstrated enhanced short-term results and similar long-term outcomes compared to open surgery.
Huang et al., 2017 [61]	563	Laparoscopic operations offer the clear advantage of being minimally invasive, with a low incidence of postoperative complications, being a viable option for treating localized gastric GISTs.
Raiter et al., 2021 [62]	21	The hybrid technique, integrating endoscopic resection and endoluminal suturing, presents a potential alternative for managing gastric GISTs with large sizes and high MP (muscularis propria) connection grades (type > I).
Fu et al., 2016 [51]	85	Imatinib adjuvant therapy does not yield significant benefits over three years in patients with intermediate risk of GISTs.
Nakanishi et al., 2015 [63]	22	When carefully indicated, laparoscopic resection for gastric GISTs is a safe and less invasive procedure compared to open surgery.
Zhang et al., 2023 [16]	18	Endoscopic resection of gastric GISTs, even those measuring up to 5 cm in diameter, demonstrates technical feasibility for both intraluminal and intra- and extraluminal growth types. Additionally, this approach can effectively manage tumors up to 7 cm in diameter.
Hechtman et al., 2015 [64]	260	An increased mitotic rate in gastric GISTs (≥5/50 HPF) was associated with the occurrence of secondary malignancies, regardless of the timing.
Nishida et al., 2018 [65]	665	In clinical practice, GISTs with tumor rupture have been identified in a subset of cases. These tumors typically display aggressive characteristics, such as larger size and higher mitotic count, irrespective of when the rupture occurred. Consequently, they present a poor prognosis even with the advent of imatinib therapy.
Yang et al., 2019 [19]	4224	Liver metastasis may be independent of bone or lung metastasis and might play a more dominant role in the prognosis of gastric GIST patients with metastasis.
Kramer et al., 2015 [50]	212	A more favorable prognosis in GIST was significantly associated with patient age under 50 years and female gender.
Jumniesuk et al., 2018 [66]	76	Metastasis was associated with tumor size greater than 10 cm, non-spindle cell histology, a mitotic count exceeding 5/5 mm^2^, myxoid change, and mucosal invasion. Recurrence was linked to myxoid change.
Slavu et al., 2019 [45]	12	From a surgical perspective, these tumors should be regarded as malignant, and adherence to principles of oncological surgery is imperative. Preserving tumor integrity during dissection is crucial for ensuring the patient’s long-term prognosis.
Stanek et al., 2019 [67]	46	Minimally invasive surgical procedures offer a viable and safe alternative for treating GISTs with a diameter exceeding 5 cm.
Stanek et al., 2019 [41]	68	Laparoscopic treatment for gastric GISTs offers indisputable advantages, such as decreased blood loss, lowered complication risks, and shorter hospital stays.
Ntourakis et al., 2015 [42]	706	In addition to conventional collaborative techniques, innovative procedures such as LECS (laparoscopic endoscopic cooperative surgery), LAEFR (laparoscopic-assisted endoscopic full-thickness resection), and NEWS (non-exposed endoscopic wall inversion surgery) show significant potential for advancing minimally invasive oncologic procedures in the future.
Liao et al., 2017 [44]	207	The laparoscopic method for managing gastric GISTs is both secure and practicable, producing oncological surgical outcomes that are widely acknowledged.

## Data Availability

Data available on request from the correspondent authors.
